# Diagnosis and Management of Patients with Connective Tissue Disease-related Fibrosing Interstitial Lung Diseases

**DOI:** 10.2174/18743064-v17-e230714-2022-26

**Published:** 2023-08-15

**Authors:** Bonnie Wang, Vivek Nagaraja

**Affiliations:** 1 Department of Internal Medicine, Division of Pulmonary and Critical Care, University of Michigan, Ann Arbor, MI 48109, USA; 2 Department of Internal Medicine, Division of Rheumatology, University of Michigan, Ann Arbor, MI 48109, USA

**Keywords:** Autoimmune disease, Connective tissue disease, Diagnosis, Fibrosis, Interstitial lung disease, Lung

## Abstract

**Background:**

Fibrotic interstitial lung disease is an important driver of morbidity and mortality in patients with connective tissue diseases (CTD). Due to the lack of prospective randomized trial data in this population, practice pattern variation exists in the management of patients with CTD.

**Case Presentation:**

This case series describes three patients, each with a different background of autoimmunity complicated by fibrotic interstitial lung disease (ILD). We review their initial presentations, follow their disease trajectories on currently available treatments, and reference forthcoming clinical trials.

**Conclusion:**

Clinical impact or potential implications. Response to immunosuppression and antifibrotic therapy is variable in patients with connective tissue disease-related fibrosing interstitial lung disease. Data from prospective clinical trials and longitudinal registry studies will conceivably provide additional insight into improving care for these patients.

## 1 INTRODUCTION

The physiologic trajectory of fibrotic interstitial lung disease (ILD) is variable and unpredictable [1]. This is unsurprising given the heterogeneity of underlying connective tissue disease (CTD), diverse radiologic patterns, and the lack of prospective randomized data on treatment modalities outside of systemic sclerosis-associated ILD. We discuss three cases, illustrating diagnostic features, monitoring, and management strategies for patients with CTD- related fibrosing ILD.

## 2 CASE PRESENTATION

### 2.1 Case 1

**Fig. (1):**
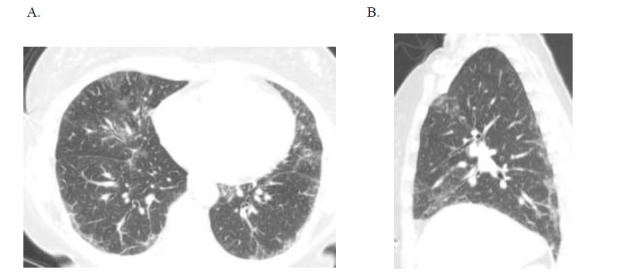
Case 1: Nonspecific interstitial pneumonitis in a 51-year-old female with autoimmune features clinically. (**A**) Axial and (**B**) sagittal HRCT images demonstrate primarily ground glass opacities with minimal reticulation, predominantly subpleural in the distribution in the mid to lower lungs; note there is no bronchiectasis or honeycombing.

**Fig. (2):**
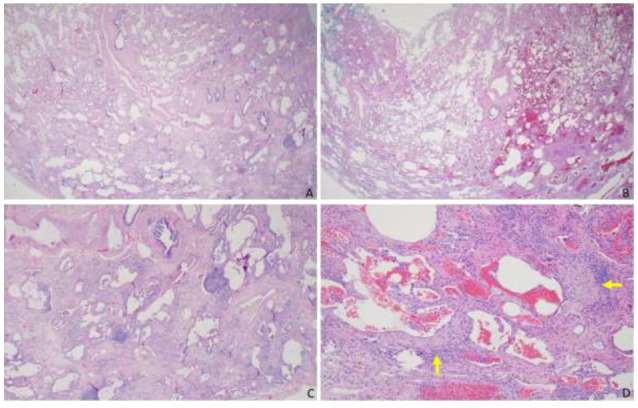
Lung wedge biopsy from the same patient in Fig. (**1**) from the right lower lobe. (**A**) and (**B**): Interstitial fibrosis has a predilection to the peripheral subpleural lung without the tissue-destructive scarring and honeycomb change characteristic for usual interstitial pneumonia (UIP) (magnification: 20x). (**C**): The accompanying inflammation is mild and composed of main lymphocytes with occasional lymphoid aggregates without secondary germinal centers (magnification: 40x). (**D**): Scattered subepithelial fibroblast foci (yellow arrow) are noted (magnification: 100x).

A 51-year-old female initially presented with a 5-day history of fever, generalized myalgia, and fatigue. She endorsed persistent throat clearing with phlegm that progressed to dyspnoea on exertion with incline. She noticed swelling in her fingers and difficulty taking off her wedding band. A few months later, she experienced triphasic Raynaud's pheno- menon, tingling and pain in the fingertips and toes, and a facial malar rash. Her exam was notable for bibasilar crackles, puffy fingers without synovitis, and clubbing. Serologies revealed an antinuclear antibody (ANA) 1:320 (speckled pattern), anti-SS-A 52 kilodaltons, rheumatoid factor 80 (normal is less than 14), and anti-PL-12. Her forced vital capacity (FVC) was 2.16 liters (61% predicted), total lung capacity (TLC) was 3.36 liters (68% predicted), and the diffusing capacity of the lung for carbon monoxide (DLCO) was 11.4 ml/min/mmHg (50% of predicted). High-resolution computed tomography (HRCT) demonstrated a fibrotic nonspecific interstitial pneumonitis (NSIP) pattern, which may represent usual interstitial pneumonia (UIP) or NSIP histologically, including CTD-ILD (Fig. **1**). Subsequent surgical lung biopsy showed unclassi-fiable chronic interstitial pneumonia with fibrosis (Fig. **2**). Discussion at our multidisciplinary ILD conference led to a diagnosis of undifferentiated forms of connective tissue disease (in the anti-synthetase disease spectrum) and a provisional classification of interstitial pneumonia with autoimmune features (IPAF) with fibrotic nonspecific ILD.

### 2.2 Case 2

**Fig. (3):**
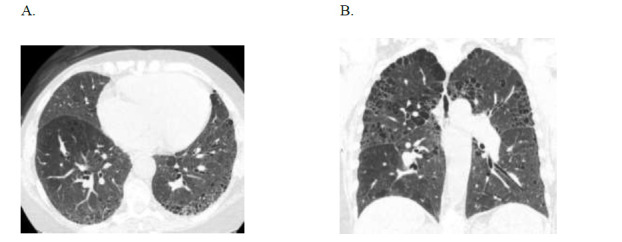
Case 2: Usual interstitial pneumonitis in a 71-year-old female with rheumatoid arthritis. (**A**) Axial and (**B**) coronal HRCT images demonstrate primarily subpleural honeycombing along both the costal and fissural pleural surfaces and mild biapical paraseptal emphysema.

A 71-year-old female with a history of breast cancer status post lumpectomy and radiation, Factor V Leiden deficiency, and prior history of smoking, initially presented with bilateral hand pain, stiffness, and swelling in 2017. Serologies were positive for ANA 1:1280, anti-double-stranded DNA, and elevated rheumatoid factor of 544. She was diagnosed with seropositive rheumatoid arthritis (RA) using the 2010 American College of Rheumatology/European League Against Rheumatism classification criteria. She was treated with steroids for three months with improvement in joint symptoms and then transitioned to sulfasalazine. Six months later, she was hospitalized for shortness of breath, productive cough, and wheezing, which improved with ipratropium and albuterol nebulisation, steroids, and antibiotics, and discharged with supplemental oxygen. At the pulmonary clinic 15 months later, she had bibasilar crackles, clubbing, and trace peripheral oedema. Her FVC was 2.75 liters (95% predicted), TLC 4.62 liters (104% predicted), and DLCO 5.24 ml/min/mmHg (25% predicted), a Pseudonormalisation pattern with a severely impaired gas exchange that complemented the severe bilateral usual interstitial pneumonia pattern and emphysema on HRCT (Fig. **3**) and co-morbid pulmonary hypertension contributing to her low DLCO. The consensus diagnosis at the multidisciplinary ILD conference was RA-associated UIP.

### 2.3 Case 3

**Fig. (4):**
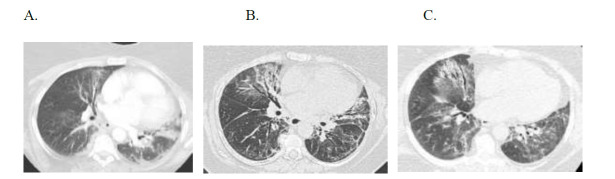
Case 3: Temporal evolution of fibrotic nonspecific interstitial pneumonitis in a 66-year-old female with an overlap of primary Sjogren’s syndrome and anti-synthetase syndrome. Axial HRCT images in (**A**) April 2017, (**B**) August 2017, and (**C**) September 2018 demonstrate basilar predominant ground glass opacities which evolved to include bronchiectasis and septal lines in the areas of ground glass four months, later indicating evolution from a primarily inflammatory process to fibrosis. 13 months later, there has been a further increase in the extent of disease with a larger percentage of abnormal lung and new ground glass opacity.

A 66-year-old woman with gastroesophageal reflux disease status post-Nissen fundoplication, with no smoking history, longstanding sicca symptoms, and a positive anti-SSA initially presented with progressive dyspnoea and dry cough. She received multiple courses of antibiotics without improvement, and HRCT revealed an NSIP pattern (Fig. **4**). Serologies were positive for ANA and anti-SSA. She had a restrictive ventilatory defect with impaired gas exchange, FVC 2.87 liters (50% predicted), TLC 2.29 liters (50% predicted), and DLCO 6.4 mL/min/mmHg (31% predicted). Oxygen assessment demonstrated a 6-liter oxygen requirement during ambulation. She experienced gastrointestinal side effects of mycophenolate mofetil (MMF) and was switched to mycophenolate sodium.

Several weeks later, she was admitted for progressive dyspnoea, new pruritic rash on her shoulders, abdomen, and back, low-grade fever, night sweats, 20-30 pound weight loss with poor appetite, myalgia, and difficulty climbing stairs. Physical examination demonstrated diffuse crackles, and synovitis in many distal and proximal interphalangeal joints bilaterally with decreased range of motion, clubbing, and 4/5 proximal muscle strength in the major large muscle groups of the upper and lower extremities. Notable labs included creatine phosphokinase 2,311 (26-180 IU/L), aldolase 40 (1-7 IU/L), aspartate aminotransferase 97, and alanine aminotransferase 90. Repeat serological studies showed anti-Jo-1 and anti-SSA 52 kilodalton antibodies. Left flank skin biopsy showed superficial perivascular and interstitial infiltration with eosinophils and neutrophils. Labial minor salivary gland biopsy showed fibrotic and atrophic tissue with moderate lymphoplasmacytic inflammation. Left vastus lateralis biopsy showed mild fiber size with rare angulated small fibers. She was diagnosed with an overlap of primary Sjogren’s syndrome and anti-synthetase syndrome and started on 1 mg/kg IV methylprednisolone (then transitioned to tapering oral prednisone) and monthly intravenous cyclophosphamide. Her course was complicated by sigmoid diverticulitis with microabscess; mycophenolate sodium was discontinued. Upon recovery, she received intravenous immunoglobulin (IVIG) infusion (6-doses, 2 g/kg) alternating with cyclophosphamide every two weeks. She reported improved muscle strength and activity levels with decreasing oxygen requirements throughout the infusion. FVC and DLCO improved, and muscle enzymes normalized. She had another flare-up of ILD and myositis requiring 6 months of monthly IV cyclophosphamide. She was eventually transitioned to a combination of mycophenolate sodium, 6- monthly intravenous rituximab, 4-weekly IVIG, and low-dose prednisone.

## 3 RESULTS AND DISCUSSION

### 3.1 Interstitial Pneumonia with Autoimmune Features

IPAF is a spectrum of diseases characterized by HRCT or surgical lung biopsy evidence of interstitial pneumonia with a suggestion of CTD based on information from three domains (extra-pulmonary autoimmune signs/symptoms, circulating autoantibodies, and/or radiologic or histologic patterns), but not definitive for CTD [2]. The ERS/ATS Task Force on Undifferentiated Forms of CTD–Associated Interstitial Lung Disease proposed IPAF classification criteria towards standardizing nomenclature [2]; one feature each in two of the three domains must be met. Case 1 has the features in all three domains: Raynaud’s phenomenon, positive antinuclear antibody 1:320 (speckled pattern), rheumatoid factor, anti-SS-A, anti-PL-12, and fibrotic NSIP HRCT patterns, with alternate interstitial pneumonia etiologies excluded. It is important to note that IPAF is a research classification and not a clinical diagnosis.

Currently, there are no specific recommendations on the use of immunomodulatory and/or anti-fibrotic therapies for IPAF; prospective studies on natural history and management strategies are needed [2].

In Case 1, MMF was started with a goal dose of 2-3 grams daily, given her degree of restriction and impaired gas exchange [3]. There are no guidelines for disease monitoring. Our practice is clinical assessment and spirometry every 3-6 months with follow-up imaging if any, worsening. If progression develops, we would add nintedanib based on the presence of dense collagen deposition in fibroblast foci on her lung biopsy and the INBUILD trial results, which showed slower FVC decline in patients with fibrosing ILD on nintedanib [4]. It is essential for clinicians to be aware of IPAF, although not a clinical diagnosis. It allows for the inclusion of additional patients who may benefit from careful monitoring by a rheumatologist to assess for progression to a recognizable CTD and treatment of ILD.

### 3.2 Rheumatoid Arthritis-associated ILD

In patients with RA, the following factors are strongly associated with RA-ILD development: advanced age, cigarette smoking history, male gender, rheumatoid factor titer, anti-cyclic citrullinated protein antibody titer, and high disease activity [5]. Clinical predictors of impairment or mortality include age, smoking history, severe disease at the presentation by baseline, FVC, FVC decline, and a UIP pattern on HRCT [6, 7]. Potential stabilisation options beyond steroid therapy derived from retrospective or observational studies include intravenous rituximab [8, 9], subcutaneous abatacept [7, 10], azathioprine, MMF [3], and tocilizumab [7]. Patients with RA-ILD have decreased survival; retrospective studies estimate a median survival after ILD diagnosis of 2.6 to 7.8 years [11]. Case 2 had many poor prognostic factors, including older age, smoking history, and HRCT UIP pattern. She was transitioned to intravenous rituximab infusion [1000 milligrams; day 1 and day 15] every six months, resulting in improved joint disease and stable lung disease with lung function and supplemental oxygen requirement. The results of TRAIL1 (NCT02808871), a phase II, randomized, placebo-controlled study of pirfenidone in 123 patients with RA-ILD have recently been published [12]. The trial was terminated early and was underpowered to detect a difference between treatment groups in the primary endpoint (decline in FVC % predicted ≥10% or death over 52 weeks). However, pirfenidone reduced the rate of decline in FVC (mL/year) over 52 weeks by 55% *versus* placebo, with an adverse event profile similar to that observed in previous trials. We await the results of APRIL (NCT03084419), a phase 2 trial investigating the safety of abatacept in RA-ILD patients with a primary endpoint of FVC change in 28 weeks.

### 3.3 Myositis-associated ILD

Idiopathic inflammatory myopathies are characterized by skeletal muscle weakness and inflammation. Major subtypes associated with ILD include dermatomyositis, polymyositis, anti-synthetase syndrome, and rheumatic overlap syndrome. Observed clinical phenotypes vary from asymptomatic to subacute/chronic to acute, more severe, and rapidly progressive disease-associated anti-MDA-5 antibodies [13]. In the setting of anti-Jo 1 positive anti-synthetase syndrome, the co-existence of anti-SSA 52 can be associated with more severe ILD, decreased remission rates with therapy, increased risk of malignancy, and decreased survival [14]. Treatment options for myositis-associated ILD include MMF, azathioprine, methotrexate, cyclosporine, rituximab, cyclophosphamide, and IVIG [15, 16]. In Case 3, the disease trajectory manifested as active myopathy and ILD exacerbation, with suspected respiratory muscle weakness. Her lung disease responded well to cyclophosphamide therapy, and she completed 12 doses. Her FVC increased to 1.82 liters (70% predicted) and DLCO 9.67 mL/min/mmHg (49% predicted). Oxygen needs to be decreased to 2 liters of supplemental oxygen on exertion, and inflammatory myopathy is in remission. We look forward to myositis-related ILD clinical trial results, including RECITAL (NCT01862926), myositis subgroup analyses of rituximab *versus* cyclophosphamide for changes in FVC, and ATtackMy-ILD (NCT0321592 - studying the safety and tolerability of abatacept for anti-synthetase syndrome-associated ILD).

### 3.4 Progressive Fibrosing ILD

Autoimmune ILDs can be associated with progressive fibrosing ILDs, defined by increased fibrosis on chest imaging, lung function decline, and/or worsening pulmonary symptoms. While establishing the type of underlying autoimmune disease is important for immunosuppression decisions, subgroup analyses by ILD diagnosis in the INBUILD trial suggest that nintedanib slowed the annual rate of FVC decline across all ILD groups and regardless of type of fibrotic pattern on HRCT [17]. While pirfenidone has an acceptable safety and tolerability profile in patients with fibrosing ILD, more studies are needed to determine the therapeutic benefit [18].

## CONCLUSION

These cases illustrate a heterogenous selection of patients with fibrosing ILD. Diagnostic dilemmas are not uncommon and highlight the need for serial follow-up and a multidisciplinary approach (Rheumatology, Pulmonology, Radiology, Pathology). Variable responses to treatment are common, and additional prospective randomized clinical trials combined with longitudinal registry data will be essential for the advancement of patient care.

## AUTHORS’ CONTRIBUTIONS

BRW and VN participated in the conception and design, analyzed the data, and drafted, revised, and approved this final version.

## Data Availability

The data and supportive information are available within the article.

## LIST OF ABBREVIATIONS

ANA: = Antinuclear Antibody
CTD: = Connective Tissue Disease
**DLCO**: = Diffusing Capacity of the Lung for Carbon Monoxide
FVC: = Forced Vital Capacity
**HRCT**: = High-resolution Computed Tomography
ILD: = Interstitial Lung Disease
**IPAF**: = Interstitial Pneumonia with Autoimmune Features
**IVIG**: = Intravenous Immunoglobulin
**MMF**: = Mycophenolate Mofetil
**NSIP**: = Non-specific Interstitial Pneumonitis
RA: = Rheumatoid Arthritis
TLC: = Total Lung Capacity
UIP: = Usual Interstitial Pneumonia

## STANDARDS OF REPORTING

CARE guidelines have been followed.
